# Impact of structured adherence training on healthcare professionals: a pilot study in Mexico and Thailand

**DOI:** 10.3389/fmed.2026.1758459

**Published:** 2026-02-19

**Authors:** Pattarapong Makarawate, Diego Araiza Garaygordobil, Tania Carmona-Luna, Patthanun Pruksamonthol, Andressa da Silva van der Laan

**Affiliations:** 1 Department of Medicine, Faculty of Medicine, Khon Kaen University, Khon Kaen, Thailand; 2 Queen Sirikit Heart Center of the Northeast (QSHC), Khon Kaen, Thailand; 3 Faculty of Medicine, Khon Kaen University, Khon Kaen, Thailand; 4 Coronary Care Unit, Instituto Nacional de Cardiología Ignacio Chávez, Mexico City, Mexico; 5 Medical Affairs, Cardiometabolic Division Abbott Mexico, Mexico City, Mexico; 6 Medical Affairs, Abbott Laboratories Limited, Bangkok, Thailand; 7 Abbott Product Operations AG, Allschwil, Switzerland

**Keywords:** chronic disease management, digital health, healthcare professionals training, Insight tool, medication adherence, my a:care app

## Abstract

**Background:**

Medication non-adherence is a significant global challenge, particularly in chronic disease management, and is associated with increased morbidity, mortality, and healthcare costs. Healthcare professionals (HCPs) play an important role in supporting adherence, yet often lack training and practical tools.

**Objectives:**

This pilot study evaluated the impact of a structured 6-month adherence training program-including educational sessions and self-learning, the Insight tool, and the my a:care app-on HCPs’ knowledge and practices in Mexico and Thailand.

**Design:**

A prospective, dual-center pilot study was conducted among HCPs managing dyslipidemic patients in two hospitals (Mexico: *n* = 15; Thailand: *n* = 27). The intervention included three in-person meetings (baseline, 3 months, 6 months) with lectures, resources, and repeated online questionnaires assessing knowledge, and clinical practices regarding medication adherence. Adoption and use of digital tools were also evaluated. Statistical analyses included Chi-squared and Student’s tests.

**Results:**

Both centers demonstrated significant improvements in HCPs’ awareness, knowledge, and practices related to medication adherence. Routine assessment of adherence increased from 47 to 87% in Mexico, and from 22 to 80% in Thailand. Use of the Insight tool rose from 0 to 53% in Mexico and from 11 to 96% in Thailand. Recommendation of the my a:care app increased from 33 to 100% in Mexico and from 15 to 92% in Thailand. HCPs’ confidence in addressing adherence improved, and more personalized support strategies were implemented. Baseline differences between centers reflected contextual factors such as prior resource access and local healthcare organization.

**Conclusion:**

A structured and standardized training program integrating behavioral science and digital tools improved HCPs’ knowledge and clinical practices regarding medication adherence in two culturally distinct settings. These results need further research to confirm the findings on a larger scale and in diverse healthcare environments.

## 1 Introduction

Medication non-adherence is a global challenge. The World Health Organization estimates that nearly 50% of patients do not take medication as prescribed, with approximately 30% failing to fill their first prescription (1). The current adherence rate is estimated at 50% in Mexico, and 53% in Thailand (2, 3). Despite advances in medical treatments, chronic disease management has not improved primarily because of non-adherence to medication which has significant implications for patient outcomes, such as avoidable complications, mortality, and healthcare costs (4).

Cardiovascular diseases remain the leading cause of mortality, with a significant proportion of patients failing to achieve optimal risk factor control. Studies have illustrated the inverse relationship between medication adherence and adverse cardiovascular outcomes, showing that a 20% improvement in adherence correlates with a 8% reduction in cardiovascular events and a 12% decrease in mortality (5). A simulation model conducted in Mexico, Thailand and China projected that increasing adherence to optimal levels among 1,000 patients could prevent 42 cardiovascular (CV) events in Mexico, 34 in Thailand, and 63 in China (6).

The reasons for non-adherence are multiple and complex, related to patient factors, treatment factors, socio-economic factors, healthcare professional and system-related factors (7–9). Healthcare professionals have a fundamental role to play in improving medication adherence, such as assessing the patient’s readiness to adhere, providing advice and monitoring the patient’s progress at every contact. However, many face lack of training and heavy workloads, and some may perceive that adherence management falls outside their responsibilities. Assessing the risk of non-adherence and using several interventions to optimize adherence levels—such as patient education, medication regimen management, clinical pharmacist consultation, cognitive behavioral therapies, medication-taking reminders and other – can make a difference. But to make this practice into reality, tools and resources are needed, and HCPs must have access to specific training in adherence management to design and maintain relevant approaches (10). For example, the adoption of digital technologies (medication reminders, real-time adherence tracking, HCPs interventions, educational content) can effectively support medication adherence and improve patients’ health outcomes (11). Studies have shown that patients using such technologies report higher adherence rates and improved Health-Related Quality of Life (HRQoL), as they feel more connected to their care and are less likely to miss doses of medication (12).

Few studies have followed up on HCPs training and evaluated the impact of providing support and tools to scale up HCPs on the subject. A European survey conducted among healthcare professionals evaluated whether participants were asking patients about missed doses and what behaviors they adopted to support patients with medication-taking (13). Another study assessed how healthcare professionals managed medication adherence, focusing on measurement, reporting and interventions (10). This study addresses the above gaps by evaluating the effectiveness of a structured training program for HCPs in two culturally distinct centers, Mexico and Thailand, and, to our knowledge, is the first to assess its impact on HCPs’ practice, knowledge and perceived relationship with their patients. It assesses changes in knowledge and practices over 6 months while learning medical content and integrating tools like patient risk assessment methods and coaching apps to support adherence. This dual-center approach provides valuable insights into the adaptability and impact of such interventions globally.

## 2 Method

The objective of this study was to measure the impact of a structured adherence training program on healthcare professionals’ (HCPs) behaviors toward their patients to improve treatment adherence, ultimately advancing patients’ better health outcomes. The first phase of this project was conducted as a pilot study in two centers to enable a first analysis and draw initial conclusions. It also aimed to explore how these insights could be implemented on a global scale.

### 2.1 Study design

This study consisted of three in-person meetings held at month 1 (T0), month 3 (T3), and month 6 (T6). An online questionnaire was administered at the beginning of each meeting, focusing on the knowledge and practices of HCPs regarding medication adherence. The study targeted HCPs involved in the management of dyslipidemic patients from two centers: one in Mexico and one in Thailand.

Meetings in Mexico were held between November 2023 and May 2024, while those in Thailand took place between May and November 2024. The meetings included the administration of online questionnaires, lectures, and resources on medication adherence. They were led by the ambassadors Prof. Makarawate in Thailand, and Dr. Araiza Garaygordobil in Mexico. The first meeting last 1.5 h. Participants first completed the questionnaire to assess baseline knowledge and use of resources related to medication adherence. This was followed by a lecture on medication adherence, as well as access to some videos and articles for deeper understanding. Participants also received explanations and access to two medication adherence tools: Insight, and my a:care app.

The second and third meetings were each 45 min long. Participants completed the same questionnaire, followed by a refresher lecture and Q&A session to discuss their learnings and challenges in practice and with adherence tools. The third meeting included a final session to review learnings and discuss difficulties encountered during the program.

### 2.2 Training/program content

In addition to the lectures, participants had access to three resources throughout the study: (1) Online articles and videos on improving adherence through behavioral science and technology; (2) Access to Insight tool, an adaptative instrument measuring key patient-level risk factors for adherence problems in chronic disease, based on four key pillar: Social (patient’s perception of support and impact of treatment on their social role), Psychological (self-concept, reactance and discounting of future benefits), Usage (practical and control elements) and Rational (perceptions of treatment benefits, illness severity, and perceived risk for adverse outcomes) (14); and (3) Access to the my a:care app, a mHealth application designed to address medication non-adherence using established behavioral methods. HCP could recommend the app to patients to encourage and monitor adherence. The app link adherence to self-care and provides motivational messages, health insights, challenges, and pill reminders.

### 2.3 Questionnaire

The questionnaire used in this study was developed specifically for the purpose of evaluating healthcare professionals’ knowledge, attitudes, and practices regarding medication adherence in dyslipidemia management. Items development was based on a review of the existing adherence literature addressing HCPs behavior and educational programs, as well as based on the authors expertise. Items assessing the learning of the training content delivered in the program were also added. Finally, authors from Thailand and Mexico also assess the questionnaire for relevance and cultural appropriateness. Meetings and emails discussions were carried out until final validation of the questionnaire. The questionnaire was then developed on the SurveyMonkey^®^ Platform, and diffused during the centers meeting via a QR code. Each participant had to acknowledge the privacy policy and sign a consent form before accessing the questionnaire, which took approximately 15 min to complete. The final questionnaire consisted of three parts: (1) Profiling, to collect information on respondents such as gender, time spent working in the center, occupation, number of patients seen per month, time spent per consultation and adherence-related habits; (2) Educational, to evaluate respondents’ knowledge of non-adherence and its evolution over time; and (3) Impact on clinical practice, to assess respondents’ practices and changes over time. Each item was answered using a Likert-scale to facilitate the analysis and observe changes over time.

The first questionnaire (T0) included the profiling and clinical practice section. Since the participants remained the same throughout the study, the profiling section was not repeated at T3 and T6. The T0 questionnaire was administered at the start of the project, before any interventions, to evaluate baseline knowledge and practices. The second questionnaire (T3) included a training assessment section to evaluate what participants had learned, as well as the clinical practice section, and was administered 3 months after the start of the project, before the refresher session. The third questionnaire (T6) was identical to the second, and was administered 3 months after the second meeting, before the final discussion session. All responses were closed-end and self-reported, reflecting the perceptions of the respondents. Questionnaires were translated in Spanish and Thai to facilitate the understanding of the questions. The English questionnaire and privacy policies are available as Supplementary materials.

### 2.4 Participants

The two selected centers were hospitals with no previous experience in adherence programs. Each project ambassador selected one center, based on the following criteria: at least 15 HCPs enrolled (no maximum), all from the same hospital or clinic (can be from different units) managing patients with dyslipidemia (cardiologists, nephrologists, endocrinologists, internists, nurses, pharmacists), able to attend to follow-up meetings every 3 months for 6 months, and open to implementing new practices in their daily consultations. Email and personal invitations were shared within the two centers, and HCPs willing to participates could register by contacting their respective ambassadors. All participants agreed to and signed a consent form before starting the program. Responses were collected only in aggregate and anonymized. The platform complied with the RGPD policies for data protection.

In the Thailand center, a small remuneration was offered to the participants to encourage their participation.

### 2.5 Descriptive and statistical analysis

Descriptive and statistical analysis were performed. Given the pilot design of this study and the small number of participants, a 90% confidence interval (CI) was used to balance the limited sample size with the need to maintain reasonable statistical power. Using a 90% CI reduces the risk of false negatives and enhances sensitivity to detect potential trends in practice changes.

A Chi-squared test was conducted to evaluate significant differences, and for some questions, a mean test (Student’s test) was also performed to maximize the identification of potential trends. Analyses were performed using COSI v4.1 software. Some participants missed one of the follow-up meetings (T3 or T6), leading to incomplete response profiles. No imputation was performed; analyses used all available data at each timepoint, and results are presented as percentages to facilitate comparison despite varying denominators.

## 3 Results

### 3.1 Profiling of the respondents

In Thailand, 27 HCPs were enrolled, between May and November 2024. All participants (100%) were nurses, most of whom dedicated 100% of their working time to the center and conducted more than 41 dyslipidemia consultations per month. On average, they saw 54 patients per month, with consultations lasting 15 to 20 min. Two participants reported using methods to assess the risk of non-adherence prior to the training (health behavior assessment forms).

In Mexico, 15 HCPs were enrolled, between November 2023 and May 2024. 60% of participants were cardiologists, 40% were general practitioners, and 80% were men. Most participants worked full-time at the study center and conducted more than 41 dyslipidemia consultations per month, for an average number of patients seen per month of 62, with average consultation durations of 16–30 min. Four participants reported prior use of methods to assess non-adherence risk (online cardiovascular risk scales, or direct questions on adherence, side effects, accessibility, and dosage of the treatment).

### 3.2 Training assessment

This section evaluated participants’ learning resulting from the training and self-study of educational materials. Overall, participants assimilated definitions from WHO and OECD reports (1, 4), although recalling the phases of adherence and phases of treatment adherence remained challenging. Both groups (Thailand and Mexico) showed increased correct responses regarding stakeholders’ involvement, with high accuracy rate by the end of the program. Nearly all participants believed by the end of the program that behavioral science can support adherence (14/15 in Mexico, and 27/27 in Thailand).

### 3.3 Impact on clinical practices

#### 3.3.1 Mexico results

##### 3.3.1.1 Adherence awareness

At T0 and T3, 100% (15 and 14 respondents, respectively) of participants rated adherence as critical to be addressed in clinical practice (score 6). However, by T6, 87% (*n* = 13) maintained this highest rating, while 13% (2 participants) rated it slightly lower, with no statistically significant differences across time points. The majority also believed that non-adherence could impact patients’ lives, with significant increase in the mean test between T3 and T6. The proportion of participants who strongly agreed that adherence can fluctuate over time increased from 33% at T0 to 64% at T3 and 67% at T6 (5, 9 and 10 respondents, respectively). While Chi-squared tests showed no significant differences at individual time points, mean tests revealed significant differences between T0 and both T3 and T6. Regarding the role of HCPs in patient adherence, 67% (10 participants) were convinced at T0, increasing to 80% or more at T3 (13 participants) and T6 (12 participants), with a significant difference between T0 and T3 on mean test.

Access to resources highly improved: 47% (*n* = 7) had access at the survey’s outset (sores 4–6), with only 7% (*n* = 1) having daily resources. After 3 months, daily resources rose to 57% (*n* = 8), and by 6 months, 80% (*n* = 12) maintained access (score 4–6), with significant differences between T0 and T6 on the mean test. Knowledge of reasons for non-adherence increased from 53% (*n* = 8) at baseline to 79% (*n* = 12) at 3 months and remained at 73% (*n* = 11) at 6 months, with significant differences in the mean test between T0 and both T3 and T6.

Accordingly, the proportion of participants addressing adherence daily with patients rosed from 67% at T0 to 100% at 3 months (scores 4–6, 10 and 14 participants, respectively), though no statistical differences were observed across time points. Finally, after 3 months, participants’ confidence in addressing non-adherence with patients increased from 47 to 64%, reaching 100% by 6 months (no statistical differences, 7, 9 and 15 participants, respectively). Results are detailed in Supplementary Table 1.

##### 3.3.1.2 Adherence support

At the survey’s outset, 47% of participants (*n* = 7) responded with high scores (4–6) on knowing how to assess levels of non-adherence and identifying underlying causes. This increased to 64% at 3 months (*n* = 9) and 87% at 6 months (*n* = 13) with a significant increase in higher scores (5–6) between T0 and T6. The median assessment of dyslipidemic patients increased from 41–60% at T0 and T3 to 61–80% at T6, with significant improvement in the mean percentage between T0 and T6.

No participants were using the Insight tool (the adaptative instrument measuring key patient-level risk factors for adherence problems in chronic disease) prior the beginning of the program; by 6 months more than half of them (53%, *n* = 8) were using it, with significant increase. The average number of dyslipidemic patients seen monthly was 62, with a statistically lower number of patients screened as high-risk at T6 compared to T3. Moreover, initially 47% (*n* = 7) of participants took no additional actions to address non-adherence, and after 6 months the vast majority implemented measures (*n* = 14), with a significant increase of the Chi-squared in recommending tools between T0 and T6.

Initially, no respondents rated above 3 the question “does assessing the level of risk help you in your conversations with patients about treatment adherence?”, but by 3 and 6 months, over half (*n* = 8) found it beneficial in their conversations with patients, with statistical differences in the mean test between T0 and both T3 and T6. Also, over 93% agreed that understanding causes of non-adherence helps tailor personalized services from the beginning, reaching 100% at 6 months (no significant differences *n* = 14 and 15, respectively).

Finally, high scores (4–6) on following instructions or tips to improve communication increased from 26 to 78% and reached 94% by T6 (*n* = 4, 11 and 14, respectively), with a significant increase in the mean test between T0 and both T3 and T6. As for the my a:care app (a mHealth application designed to address medication non-adherence using established behavioral methods), if 67% (*n* = 10) of participants did not recommend it at T0, the number dropped to 7% at T3 (*n* = 1), and by T6 all participants had recommended it. Moreover, 73% recommended the app based on the Insight tool and risk levels (*n* = 11), with significant increases in recommendations between T0–T3 and T0–T6 months. Results are shown in Supplementary Table 2.

#### 3.3.2 Thailand results

##### 3.3.2.1 Adherence awareness

At baseline, nearly all participants rated adherence as critical (score 6) to be addressed in clinical practice (*n* = 24). While the most maintained this rating, 27% (*n* = 9) and 24% (*n* = 12) rated it lower at T3 and T6 respectively, with a significant decrease in score 6 between T0 and T3. Most agreed non-adherence can have an impact on patient lives, though two participants at T6 still considered it not important (no statistical difference). Perceptions that adherence fluctuates over time and that HCPs can influence patient adherence remained stable, with no significant changes.

Access to resources improved from 33% at baseline (*n* = 9) to 89% at 3 months (*n* = 23) and 96% (*n* = 24) at 6 months, with significant decreases in scores 1–3 and increases in score 5 between T0 and both T3 and T6, confirmed by mean tests for all timings. For reasons for non-adherence (scores 4–6), only 52% of participants (*n* = 14) knew about it at T0 to 88% at 6 months (*n* = 22), with significant improvement seeing by mean tests.

Additionally, participants addressed adherence routinely from 19, to 31 and 44% at T6 (*n* = 5, 8 and 11, respectively), with significant improvements in score 6 and mean test between T0 and T6. Finally, after 3 months of training, participants’ confidence in addressing non-adherence with patients (scores 5–6) increased from 22 to 48%, and reached 68% by 6 months (*n* = 6, 13 and 18), though without statistical significance. Results are detailed in Supplementary Table 1.

##### 3.3.2.2 Adherence support

Confidence in assessing non-adherence and identifying causes increased from 22% at baseline to 73% at 3 months and 80% at 6 months (*n* = 6, 19 and 20, respectively), with significant decreases in score 1 and increases in score 5 between T0 and both T3 and T6. The median percentage of dyslipidemic patients assessed remained 41–60% across all time points, with a mean test statistically higher between T0 compared to T6. Only two participants were using the Insight tool at the survey’s baseline, and by 6 months all but one were using it, with significant increases in “yes” responses and decreases in “no” responses between T0 and both T3 and T6. If 19% of participants were not taking any other actions to address non-adherence at T0 (*n* = 5), after 6 months, the vast majority had begun implementing measures, with significant increases in recommending using a tool between T0 and both T3 and T6. Moreover, assessing the level of risk helped daily 50% of respondents at T0 and up to 100% by T6, with all participants believing that assessing the level of risks is beneficial in their conversations with patients, with statistical differences in the mean test between T0 compared to T3 and T6. Also, all participants agreed from baseline that understanding causes of non-adherence helps tailor personalized services (no significant differences).

Following instructions or practical tips to improve communication also had an increase in high scores over time (4–6), from 42 to 85% at T3, and up to 100% by T6 (*n* = 6, 19 and 20 respectively), with significant increases in high scores between T0 and both T3 and T6.

Finally, recommendation of the my a:care app increased substantially: if 85% of participants never recommended it at T0 (*n* = 22), only 8% remained at T6 (*n* = 2). Additionally, 72% recommended the app based on Insight tool risk level, with significant increases in “yes” responses and recommendation “to all high-risk patients” between T0 and both T3 and T6.

The number of participants recommending the app to 50 patients or more patients also increased significantly. Results are available in Supplementary Table 2.

## 4 Discussion

This study aimed to evaluate the impact of an educational program on HCPs’ knowledge and practices regarding medication adherence over a 6-month period. The program—which included structured training, the Insight tool, and the my a:care app—appeared to have a positive impact on HCPs’ awareness, knowledge, and practices related to medication adherence in both Mexico and Thailand. Improvements observed at the 3 months mark were generally maintained or further enhanced at the 6-month follow-up, suggesting a long-term impact of the intervention. Knowledge globally increased, but some notions remained difficult to recall, and some replies worsen over time illustrating the difficulties encountered by HCPs in real life: HCPs already have a lot of data to remember and not a lot of time to allocate to education and training.

The program significantly increased adherence awareness and support practices among HCPs in both Mexico and Thailand and allowed participants to gain access to resources on adherence. Both settings showed improvements in their understanding of importance of adherence, the fluctuating nature of adherence, reasons for non-adherence, and acknowledged that adherence plays an important role in chronic disease management, which is in line with the OECD report highlighting that addressing non-adherence is a key strategy to reduce the pressure on healthcare systems, and involving all stakeholder, including raising healthcare professionals awareness on the subject is important (4). While raising awareness among HCPs is the first step, patients’ behavior towards prescribers’ recommendations can differ and a low level of awareness is observed globally. A good patient-healthcare professional relationship with trust, communication and comprehension is fundamental for good adherence (10), but disparities in access to healthcare between countries and regions, like differences in knowledge of their disease, attitudes towards medication taking, beliefs that a patient holds about the treatment or the disease, and quality control among chronic diseases patients can impact levels of adherence (4, 15).

Regarding the training and tools implementation, both centers demonstrated an increased utilization of the Insight tool and recommendation of the my a:care app, with a significant increase in the use of the Insight tool to identify high-risk patients. The Insight adherence profiling tool is based on the algorithm SPUR (Social, Psychological, Usage and Rational) which evaluate the risk of non-adherence of patients, allowing effective intervention by providing insights into the respective individual reasons for lack of adherence. Its model allows it to be adapted to each patient, and so reduce testing burden while generating reliable, useful information (14, 16). Also, proposing the my a:care app to patients struggling with their adherence journey (remember, motivation, tracking, games…) may offer a great support, as shown in a literature review that eHealth provides an opportunity to offer medication adherence interventions with minimal effort from health care providers whose time and resources are limited, and that this type of intervention can be effective in improving medication adherence (17). Mobile applications in particular have been effective in chronic disease management by empowering patients to take an active role in monitoring their health, leading to better outcomes and higher satisfaction (12). A meta-analysis of 14 randomized controlled trials involving 1,785 participants showed that mobile app interventions significantly improved medication adherence in patients with chronic diseases, with 84.5% of respondents felt that apps improved their independence in managing medications and 86.8% believed apps could improve effective communication between doctors and patients (18).

Moreover, the program had a positive impact on HCPs adherence-related clinical practices. It was associated with improvements in HCPs’ confidence in addressing adherence with patients, their routine assessment of adherence levels, and the implementation of interventions to support adherence. Results also showed more HCPs assessing non-adherence at the end of the study compared to the beginning, and providing more personalized services to each patient. Furthermore, both centers agreed on the need for patients’ personalized management and tailored interventions. These observations are in line with the European ENABLE’s key recommendations mentioning that implementation strategies and beneficial effects of digital technologies are supportive adaptative intervention to support adherence (11).

However, some differences were observed between centers. First, Mexico started with a higher adherence awareness baseline compared to Thailand. This can be explained by differences in access to resources where Mexico had also more access before the beginning of the program than Thailand. Educational background, country-specific health system and national support systems can lead to disparities between these 2 countries. For example, the Mexico Institute of Social Security (IMSS) has implemented a preventive model for non-communicable chronic diseases, with educational programs for adults risk-factors and lifestyle modifications (2), where in Asia no strategies are clearly put in place to promote medical adherence, and that the cultural backgrounds Asians were more likely to perceived medicines as being harmful (3).

These results showed that education programs, when supported by the rights tools and follow-up, are beneficial for modifying healthcare professionals’ behaviors related to adherence support, and that these improvements are maintained over time.

### 4.1 Strengths and limitations of this study

This study has several strengths. It provides an overview of how a structured training program was implemented within healthcare centers and its impact on clinical practice. The multicenter design, involving two countries from distinct socio-cultural backgrounds, strengthens the generalizability of the findings. Importantly, the training content and assessment tools were standardized across both sites, enhancing consistency and reinforcing the real-world applicability of this pilot study. The longitudinal design further allowed for the monitoring of potential changes in practices over time. However, some limitations must be acknowledged. First, the relatively small number of participants (<30) limits the statistical power and requires cautious interpretation of the results. Second, the absence of a control group makes it difficult to directly attribute observed changes to the training intervention alone. Third, declarative (self-reported) data may be subject to reporting biases, and potential selection bias cannot be excluded, as participants may have been more motivated or receptive to change. Additionally, the Hawthorne effect—behavior modification due to being observed—may have influenced responses. Finally, in the Thai center, only nurses participated due to the local care structure, which may have introduced some bias in responses to some questions.

### 4.2 Future research

This program may be extended to other centers, led by an ambassador within each center, using the same training content, program structure, and questionnaire, to confirm these results. Future studies should aim to build on these preliminary findings by including larger sample sizes and extending follow-up periods to better evaluate the long-term impact and sustainability of training-induced changes. Interventional trials could also help determine the effectiveness of specific components of the training. Further research should also explore whether these changes in HCPs practice translate into improved patient outcomes. In addition, studies are needed to assess how this type of personalized program could be developed in larger scale and implemented in general practices in diverse healthcare settings.

## 5 Conclusion

This study provides preliminary evidence that structured and standardized training may contribute to modifying healthcare professionals’ behaviors related to adherence support, and that changes are maintained over time. Variability in initial levels of awareness and local organization between both centers highlight the need to consider context-specific factors during shaping interventions. These results need further research to confirm the findings on a larger scale and in diverse healthcare settings.

## Data Availability

The raw data supporting the conclusions of this article will be made available by the authors, without undue reservation.
