# Supplementary material for: Impact of structured adherence training on healthcare professionals: a pilot study in Mexico and Thailand
Source: Front Med (Lausanne). 2026 Feb 19;13:1758459. doi: 10.3389/fmed.2026.1758459 (PMC12960191; doi:10.3389/fmed.2026.1758459)
Supplement: Supplementary file 1 [file Table_1.docx]

**Table 1**: Adherence awareness (impact of educational tools)

Score 1 was the lower score, mentioning the item as not important. Score 6 was the higher score, presenting the item as important/crucial. Statistical differences with the Chi-squared test are represented in blue.

|  | **MEXICO (N= 15 at T0 and T6, and N=14 at T3)** | | | | | | | | | | | | | **THAILAND (N=27 at T0, 26 at T3, and 25 at T6)** | | | | | | | | | | | |
| --- | --- | --- | --- | --- | --- | --- | --- | --- | --- | --- | --- | --- | --- | --- | --- | --- | --- | --- | --- | --- | --- | --- | --- | --- | --- |
|  |  | **1**  n % | | **2**  n % | | **3**  n % | | **4**  n % | | **5**  n % | | **6**  n % | | **1**  n % | | **2**  n % | | **3**  n % | | **4**  n % | | **5**  n % | | **6**  n % | |
| **Do you think adherence is an important topic in your clinical practice?** | T0 | 0 | 0 | 0 | 0 | 0 | 0 | 0 | 0 | 0 | 0 | 15 | 100 | 0 | 0 | 0 | 0 | 1 | 3,7 | 2 | 7,4 | 0 | 0 | 24 | 88,9 |
|  | T3 | 0 | 0 | 0 | 0 | 0 | 0 | 0 | 0 | 0 | 0 | 14 | 100 | 0 | 0 | 0 | 0 | 2 | 7,7 | 2 | 7,7 | 5 | 19,2 | 17 | 65,4 |
|  | T6 | 0 | 0 | 0 | 0 | 0 | 0 | 0 | 0 | 2 | 13,3 | 13 | 86,7 | 0 | 0 | 0 | 0 | 1 | 4 | 3 | 12 | 3 | 12 | 18 | 72 |
| **Do you think non-adherence can have an impact on patients’ lives?** | T0 | 0 | 0 | 0 | 0 | 0 | 0 | 1 | 6,7 | 2 | 13,3 | 12 | 80 | 1 | 3,7 | 0 | 0 | 1 | 3,7 | 2 | 7,4 | 0 | 0 | 23 | 85,2 |
|  | T3 | 0 | 0 | 0 | 0 | 0 | 0 | 0 | 0 | 0 | 0 | 14 | 100 | 0 | 0 | 0 | 0 | 3 | 11,5 | 2 | 7,7 | 5 | 19,2 | 16 | 61,5 |
|  | T6 | 0 | 0 | 0 | 0 | 0 | 0 | 0 | 0 | 3 | 20 | 12 | 80 | 2 | 8 | 0 | 0 | 0 | 0 | 3 | 12 | 4 | 16 | 16 | 64 |
| **Do you think adherence can fluctuate over time for each patient?** | T0 | 0 | 0 | 0 | 0 | 0 | 0 | 1 | 6,7 | 9 | 60 | 5 | 33,3 | 0 | 0 | 0 | 0 | 1 | 3,7 | 5 | 18,5 | 12 | 44,4 | 9 | 33,3 |
|  | T3 | 0 | 0 | 0 | 0 | 0 | 0 | 0 | 0 | 5 | 35,7 | 9 | 64,3 | 0 | 0 | 1 | 3,8 | 0 | 0 | 4 | 15,4 | 14 | 53,8 | 7 | 26,9 |
|  | T6 | 0 | 0 | 0 | 0 | 0 | 0 | 0 | 0 | 5 | 33,3 | 10 | 66,7 | 0 | 0 | 0 | 0 | 1 | 4 | 7 | 28 | 10 | 40 | 7 | 28 |
| **Do you think healthcare professionals can influence patients’ adherence?** | T0 | 0 | 0 | 0 | 0 | 0 | 0 | 1 | 6,7 | 4 | 26,7 | 10 | 66,7 | 0 | 0 | 0 | 0 | 1 | 3,7 | 3 | 11,1 | 9 | 33,3 | 14 | 51,9 |
|  | T3 | 0 | 0 | 0 | 0 | 0 | 0 | 0 | 0 | 1 | 7,1 | 13 | 92,9 | 0 | 0 | 0 | 0 | 1 | 3,8 | 7 | 26,9 | 4 | 15,4 | 14 | 53,8 |
|  | T6 | 0 | 0 | 0 | 0 | 0 | 0 | 1 | 6,7 | 2 | 13,3 | 12 | 80 | 0 | 0 | 0 | 0 | 1 | 4 | 4 | 16 | 6 | 24 | 14 | 56 |
| **Do you have access to information/literature/ training on adherence?** | T0 | 1 | 6,7 | 4 | 26,7 | 3 | 20 | 5 | 33,3 | 1 | 6,7 | 1 | 6,7 | 6 | 22,2 | 3 | 11,1 | 9 | 33,3 | 4 | 14,8 | 2 | 7,4 | 3 | 11,1 |
|  | T3 | 1 | 7,1 | 1 | 7,1 | 4 | 28,6 | 1 | 7,1 | 2 | 14,3 | 5 | 35,7 | 0 | 0 | 1 | 3,8 | 2 | 7,7 | 6 | 23,1 | 9 | 34,6 | 8 | 30,8 |
|  | T6 | 0 | 0 | 1 | 6,7 | 2 | 13,3 | 5 | 33,3 | 3 | 20 | 4 | 26,7 | 0 | 0 | 0 | 0 | 1 | 4 | 3 | 12 | 8 | 32 | 13 | 52 |
| **Do you know the reasons why patients might not adhere to treatment?** | T0 | 0 | 0 | 0 | 0 | 7 | 46,7 | 7 | 46,7 | 1 | 6,7 | 0 | 0 | 2 | 7,4 | 1 | 3,7 | 10 | 37 | 9 | 33,3 | 3 | 11,1 | 2 | 7,4 |
|  | T3 | 0 | 0 | 1 | 7,1 | 2 | 14,3 | 4 | 28,6 | 5 | 35,7 | 2 | 14,3 | 1 | 3,8 | 0 | 0 | 5 | 19,2 | 5 | 19,2 | 11 | 42,3 | 4 | 15,4 |
|  | T6 | 0 | 0 | 0 | 0 | 4 | 26,7 | 3 | 20 | 5 | 33,3 | 3 | 20 | 0 | 0 | 1 | 4 | 2 | 8 | 7 | 28 | 8 | 32 | 7 | 28 |
| **Do you address adherence during consultations with your patients?** | T0 | 0 | 0 | 3 | 20 | 2 | 13,3 | 2 | 13,3 | 5 | 33,3 | 3 | 20 | 1 | 3,7 | 4 | 14,8 | 5 | 18,5 | 6 | 22,2 | 6 | 22,2 | 5 | 18,5 |
|  | T3 | 0 | 0 | 0 | 0 | 0 | 0 | 6 | 42,9 | 3 | 21,4 | 5 | 35,7 | 0 | 0 | 0 | 0 | 4 | 15,4 | 6 | 23,1 | 8 | 30,8 | 8 | 30,8 |
|  | T6 | 0 | 0 | 0 | 0 | 3 | 20 | 2 | 13,3 | 4 | 26,7 | 6 | 40 | 0 | 0 | 0 | 0 | 3 | 12 | 5 | 20 | 6 | 24 | 11 | 44 |
