# Supplementary material for: Impact of structured adherence training on healthcare professionals: a pilot study in Mexico and Thailand
Source: Front Med (Lausanne). 2026 Feb 19;13:1758459. doi: 10.3389/fmed.2026.1758459 (PMC12960191; doi:10.3389/fmed.2026.1758459)
Supplement: Supplementary file 2 [file Table_2.docx]

**Table 2** : Adherence support

Score 1 was the lower score, mentioning the item as not important. Score 6 was the higher score, presenting the item as important/crucial. Statistical differences are represented in blue. Light shade of blue illustrated a statistical difference only with T0, but not with the other timings. For example, on the first question, we have a statistical increased of score 5 in Thailand between T0-T3 and T0-T6, but T3 and T6 have no statistical difference.

|  | | **MEXICO (N= 15 at T0 and T6, and N=14 at T3)** | | | | | | | | | | | | | **THAILAND (N=27 at T0, 26 at T3, and 25 at T6)** | | | | | | | | | | | | |
| --- | --- | --- | --- | --- | --- | --- | --- | --- | --- | --- | --- | --- | --- | --- | --- | --- | --- | --- | --- | --- | --- | --- | --- | --- | --- | --- | --- |
|  |  | **1**  n % | | **2**  n % | | **3**  n % | | | **4**  n % | | **5**  n % | | **6**  n % | | **1**  n % | | **2**  n % | | **3**  n % | | **4**  n % | | **5**  n % | | **6**  n % | |  |
| **You know how to assess levels of non-adherence in your patients and identify the underlying causes** | T0 | 1 | 6,7 | 5 | 33,3 | 2 | 13,3 | | 5 | 33,3 | 2 | 13,3 | 0 | 0 | 9 | 33,3 | 4 | 14,8 | 8 | 29,6 | 5 | 18,5 | 1 | 3,7 | 0 | 0 |  |
|  | T3 | 1 | 7,1 | 1 | 7,1 | 3 | 21,4 | | 4 | 28,6 | 2 | 14,3 | 3 | 21,4 | 1 | 3,8 | 0 | 0 | 6 | 23,1 | 7 | 26,9 | 8 | 30,8 | 4 | 15,4 |  |
|  | T6 | 0 | 0 | 1 | 6,7 | 1 | 6,7 | | 5 | 33,3 | 4 | 26,7 | 4 | 26,7 | 0 | 0 | 1 | 4 | 4 | 16 | 7 | 28 | 7 | 28 | 6 | 24 |  |
| **Are you following any instructions or practical tips to improve your style of communication?** | T0 | 3 | 20 | 6 | 40 | 2 | 13,3 | | 2 | 13,3 | 2 | 13,3 | 0 | 0 | 4 | 15,4 | 3 | 11,5 | 8 | 30,8 | 4 | 15,4 | 4 | 15,4 | 3 | 11,5 |  |
|  | T3 | 0 | 0 | 1 | 7,1 | 2 | 14,3 | | 3 | 21,4 | 3 | 21,4 | 5 | 35,7 | 0 | 0 | 0 | 0 | 4 | 15,4 | 5 | 19,2 | 10 | 38,5 | 7 | 26,9 |  |
|  | T6 | 0 | 0 | 0 | 0 | 1 | 6,7 | | 6 | 40 | 4 | 26,7 | 4 | 26,7 | 0 | 0 | 0 | 0 | 0 | 0 | 6 | 25 | 10 | 41,7 | 8 | 33,3 |  |
|  | | **1** | | | **2** | | | | **3** | | | **4** | | | **1** | | | **2** | | | **3** | | | **4** | | |  |
| **Assessing the level of risk help you in your conversations with patients about treatment adherence** | T0 | 1 | 6,7 | | 5 | 33,3 | | | 9 | 60 | | 0 | 0 | | 3 | 21,4 | | 4 | 28,6 | | 2 | 14,3 | | 5 | 35,7 | |  |
|  | T3 | 1 | 7,1 | | 0 | 0 | | | 5 | 35,7 | | 8 | 57,1 | | 0 | 0 | | 3 | 16,7 | | 4 | 22,2 | | 11 | 61,1 | |  |
|  | T6 | 0 | 0 | | 0 | 0 | | | 7 | 46,7 | | 8 | 53,3 | | 0 | 0 | | 0 | 0 | | 2 | 28,6 | | 5 | 71,4 | |  |
|  | | **No** | | | **Explain the importance of adherence** | | | | **Discuss the reasons for non-adherence** | | | **Recommend tool** | | | **No** | | | **Explain the importance of adherence** | | | **Discuss the reasons for non-adherence** | | | **Recommend tool** | | |  |
| **Are you taking any other actions not mentioned in this questionnaire to address non-adherence?** | T0 | 7 | 46,7 | | 8 | 53,3 | | | 6 | 40 | | 1 | 6,7 | | 5 | 19,2 | | 20 | 76,9 | | 10 | 38,5 | | 1 | 3,8 | |  |
|  | T3 | 0 | 0 | | 9 | 64,3 | | | 7 | 50 | | 7 | 50 | | 2 | 7,7 | | 21 | 80,8 | | 9 | 34,6 | | 10 | 38,5 | |  |
|  | T6 | 1 | 6,7 | | 11 | 73,3 | | | 10 | 66,7 | | 10 | 66,7 | | 5 | 20,8 | | 15 | 62,5 | | 9 | 37,5 | | 10 | 41,7 | |  |
|  | | **Yes** | | | | **No** | | | | | **I don’t know** | | | | **Yes** | | | | **No** | | | | **I don’t know** | | | |  |
| **Understanding the causes of non-adherence helps you to provide personalized services that are tailored to your patients' needs** | T0 | 14 | 93,3 | | | 1 | | 6,7 | | | 0 | 0 | | | 26 | 100 | | | 0 | 0 | | | 0 | 0 | | |  |
|  | T3 | 13 | 92,9 | | | 0 | | 0 | | | 1 | 7,1 | | | 26 | 100 | | | 0 | 0 | | | 0 | 0 | | |  |
|  | T6 | 15 | 100 | | | 0 | | 0 | | | 0 | 0 | | | 24 | 100 | | | 0 | 0 | | | 0 | 0 | | |  |
| **Are you using a:care insight, Abbott’s behavioral diagnostic tool?** | T0 | 0 | 0 | | | 15 | | 100 | | |  | | | | 2 | 7,4 | | | 25 | 92,6 | | |  | | | |  |
|  | T3 | 4 | 28,6 | | | 10 | | 71,4 | | |  |  |  |  | 23 | 88,8 | | | 3 | 11,5 | | |  |  |  |  |  |
|  | T6 | 8 | 53,3 | | | 7 | | 46,7 | | |  |  |  |  | 24 | 96 | | | 1 | 4 | | |  |  |  |  |  |
| **Are you recommending my a:care app to patients who are at risk of non-adherence?** | T0 | 5 | 33,3 | | | 10 | | 66,7 | | |  | | | | 4 | 15,4 | | | 22 | 84,6 | | |  | | | |  |
|  | T3 | 13 | 92,9 | | | 1 | | 7,1 | | |  |  |  |  | 23 | 92 | | | 2 | 8 | | |  |  |  |  |  |
|  | T6 | 15 | 100 | | | 0 | | 0 | | |  |  |  |  | 24 | 100 | | | 0 | 0 | | |  |  |  |  |  |
