# Supplementary material for: Impact of structured adherence training on healthcare professionals: a pilot study in Mexico and Thailand
Source: Front Med (Lausanne). 2026 Feb 19;13:1758459. doi: 10.3389/fmed.2026.1758459 (PMC12960191; doi:10.3389/fmed.2026.1758459)
Supplement: Supplementary file 3 [file Table_3.docx]

**Supplementary 1: English questionnaire of the pilot study**

Please include your email: *mandatory** (the selected centers need to sign a “consent to use” during enrolment)*

**PART 1: PROFILING**

1. Please indicate your center:
   - Center 1 in Thailand
   - Center 2 in Thailand
   - Center in China
   - Center in Mexico
2. What proportion of your working time is spent at this center?
   - 100% (full-time)
   - 75% (¾ time)
   - 50% (half-time)
   - 25% (¼ time)
3. What is your gender?
   - Male
   - Female
   - Prefer not to say
4. What is your occupation?
   - General practitioner
   - Nurse
   - Pharmacist
   - Specialist:
     - Cardiologist
     - Nephrologist
     - Endocrinologist
     - Internist
   - Other (please specify)
5. What is the average number of patients with dyslipidemia you typically see in consultation at your center per month?
   - 0-20
   - 21-40
   - 41-60
   - 61-80
   - >81
6. What is the average time spent with each patient for a consultation?
   - <5 min
   - 5-15 min
   - 16-30 min
   - >30 min
7. Do you use any tools or questionnaires to identify the level/risk of non-adherence in your patients?
   - Yes
     - Which one(s)?
   - No
8. Do you recommend a digital coach/support to your patients to help them manage and keep track of their condition?
   - Yes
     - Which one(s)?
   - No
9. Are you familiar with the a:care program? (*Question for T0 only*)
   - Yes
     - Where did you learn about it? – Medical sales representatives, email, medical events, media, etc. Include options (sales representative, media, etc.)
   - No

**PART 2: TRAINING ASSESSMENT**

Please answer these questions after watching the a:care package training (*e.g. The global challenge of medication adherence video, and the following articles: Role of healthcare practitioners in enhancing medication adherence, strategies to improve adherence to medication, using tools to assess patient adherence, and predicting non-adherence behavior using behavioral science*).

1. What is the conclusion of the 2003 WHO report on medication adherence: (*multiple-choice answer*)
   - 30-50% medicines prescribed for long-term illness are not taken as directed
   - 30-50% medicines prescribed for long-term illness are taken as directed
   - Non-adherence is not a global problem, not all countries are affected by it
   - Non-adherence is a global problem that affects all countries
2. What are the conclusions of the OECD health paper: (*multiple-choice answer*)
   - Poor adherence contributes to 200,000 premature deaths in the EU per year
   - More than 87% patients fill their first prescription
   - At treatment initiation, 50-70% are consistently adherent
   - At treatment initiation, 70-80% are consistently adherent
3. What are the 3 phases of adherence:
   - Uptake, implementation, and persistence
   - Prescription, fill-in, and follow-up
   - Consciousness, willingness, and actions
4. What are the 3 phases of treatment adherence?
   - Capability, opportunity, motivation
   - Consultation, daily life, mindset
   - Habits, socioeconomic situation, medical follow-up
5. Who are the stakeholders that should address adherence with patients? (*multiple-choice answer*)
   - Physicians
   - Nurses
   - Pharmacists
   - Dieticians
   - Friends
   - Influencers
6. Which are the factors that impact non-adherence? (*multiple-choice answer*)
   - Socio-economic factors
   - Disease-related factors
   - Patient-related factors
   - Therapy-related factors
   - Healthcare system-related factors
   - Physician-related factors
7. Non-adherence contributes to an increase in: (*multiple-choice answer*)
   - Mortality
   - Morbidity
   - Health costs
   - Quality of life
   - Wellbeing
8. Do you think behavioral science can support you in addressing adherence? *(Scale “strongly agree”, “agree”, “partially agree”, partially disagree”, “disagree”, and “strongly disagree”)*

**PART 3: IMPACT ON CLINICAL PRACTICE**

1. ***Adherence awareness*** *(impact of educational tools)*
2. Do you think adherence is an important topic in your clinical practice? *(Scale between 1 to 6 “adherence is not important – adherence is critical to be addressed in clinical practice”)*
3. Do you think non-adherence can have an impact on patients’ lives? *(Scale between 1 to 6 “adherence has 0 impact – adherence has a crucial impact”)*
4. Do you think adherence can fluctuate over time for each patient*? (Scale between 1 to 6 “strongly agree”, “agree”, “partially agree”, partially disagree”, “disagree”, and “strongly disagree”)*
5. Do you think healthcare professionals (HCPs) can influence patients’ adherence? *(Scale between 1 to 6 “HCPs can’t influence adherence at all" – "HCPs play a crucial role”)*
6. Do you have access to information/literature/training on adherence? (Scale *between 1 to 6 “0 access to anything" – "daily reading/training on the subject”)*
7. Do you know the reasons why patients might not adhere to treatment? *(Scale between 1 to 6 “No, I don’t have a clue" - "Yes, I know everything about it”)*
8. Do you address adherence during consultations with your patients? *(Scale between 1 to 6 “never address" – "routinely address”)*
   - If so, does addressing adherence with your patients comes easily to you? *(Scale between 1 to 6 “No, I don’t know what to do/say" – "Yes, very easily - I know/techniques - I’m comfortable with the subject") (this question is intended to be completed only by HCPs who replied "yes" to the last question)*
9. ***Adherence support*** *(impact of tools)*
10. ***A:CARE INSIGHT***

i. Assess non-adherence risk

1. Do you know how to assess levels of non-adherence in your patients and identify the underlying causes? *(Scale between 1 to 6 “No, never" – "Yes, daily”)*
   - If yes, what percentage of your dyslipidemic patients are being assessed over a month? (*Scale between 0% to 100%)*
2. Are you using a:care insight, Abbott’s behavioral diagnostic tool?
   - *Yes/ no*
     - If yes, over a period of a month, what percentage of screened patients would have been identified as:
       - *Very high risk – xxx %*
       - *High-risk – xxx %*
       - *Medium risk – xxx%*
       - *Low risk – xxx%*
       - *Very low risk – xxx %*

| ***Very low****: This profile suggests that there is a very low risk that the patient will not follow the doctor's recommendations.*  ***Low****: This profile suggests a low level of concern regarding the patient's adherence to the prescribed treatment plan.*  ***Medium****: This profile suggests a moderate ability of the patient to follow the prescribed treatment plan.*  ***High****: This profile indicates a high level of uncertainty about the patients’ ability to follow the prescribed treatment plan.*  ***Very high****: This profile reveals a significant concern regarding the patient's ability to comply with the prescribed treatment plan.* |
| --- |

1. Are you taking any other actions not mentioned in this questionnaire to address non-adherence? (multiple-choice answer)
   - No
   - Explain to your patients the importance of adherence for their health
   - Discuss the reasons for low adherence
   - Recommend using a tool (app, device, etc.)

ii. Reasons

1. Does assessing the level of risk help you in your conversations with patients about treatment adherence:
   - Yes/no, or rate
2. Do you think that understanding the causes of non-adherence helps you to provide personalized services that are tailored to your patients' needs?
   - Yes/No/I don’t know

iii.Instructions/ tips

1. Are you following any instructions or practical tips to improve your style of communication? *(Scale between 1 to 6 “No, I don’t know what to say/do" – "Yes, I use the insights provided by the tool on a daily basis”)*
2. ***MY A:CARE APP***
3. Are you recommending my a:care app (personal digital coach) to patients who are at risk of non-adherence?
   - *“No, never”*
   - *“Yes, to some patients based on intuition”*
   - *“Yes, to some patient based on the a:care insight profiling”*
   - *“Yes, to all high-risk patients”*
   - *“Yes, to all my patients”*
4. If yes, please specify the number of patients whom you recommended the app on the past 3-months:

| **T0 only**   1. Don’t forget to encourage your patients to stay on treatment and build healthy habits, and to demonstrate how to keep their "cube" green by accumulating consecutive "green" days.   **T3 and T6 months only**   1. After 30 days of use – is the cube still green?    - Yes, the patient continues to use it    - It depends on days; the patient does not consistently use the application    - No, the patient is no longer using application |
| --- |

**Supplementary 2: Privacy Policies**

Thank you for participating in the program measuring the **a:care impact on treatment adherence.**

The online questionnaire is a 15-minute qualitative survey that aims to collect information on Health Care Professionals' (HCP)'s perception and behavior toward patient-treatment adherence in 4 centers in China, Thailand, and Mexico.

This program focus on adherence of patients with dyslipidemia, based on HPCs' perspective.

No information collected in this questionnaire is related to patient data, and the results will be analyzed, reported, and communicated in aggregate only. We look forward to sharing the results with you as soon as they become available.

All responses are entirely confidential as all data are presented in aggregate form only. Please give considered and honest answers to the questions. Yours sincerely,

**Andressa van der Laan,** Global Medical Manager & Digital Healthcare, Abbott Products Operations AG.

**PRIVACY POLICY FOR THE a:care behavior change impact program**

Abbott acknowledges and respects the privacy of individuals. This Privacy Policy explains how we handle the personal information provided by you in this registration form. The collection, use, retention, and disclosure of your personal information is managed as detailed below:

**Data controller of personal information collected**

Abbott will operate as a Data Controller. The personal information you enter in this registration form will be shared by Abbott with its Abbott affiliate companies as necessary to fulfil the purposes described below.

**Personal information collected**

Your name, e-mail and center of exercise will be collected.

**Purpose for collecting personal information and its related use**

Your personal information is collected to send you a link to the survey and to analyze result center per center.

**Personal information disclosure to third parties**

We will not sell, share, or otherwise distribute your personal information to third parties except as this Privacy Policy provides. We may disclose your personal information to our Abbott affiliates worldwide who agree to treat it in accordance with this Privacy Policy, and always in accordance with the purposes stated above. Your personal information may also be transferred to third parties who act for or on our behalf in accordance with the purposes described above. These third parties may be located in countries or territories which may not offer the same level of data protection as the country in which you reside. They have contracted with us to use your personal information solely for the agreed purpose, not to sell your personal information to third parties, and not to disclose it to third parties except as may be required by law, as permitted by us, or as stated in this Privacy Policy.

Your personal information may also be transferred to a third party if this part of our business and the personal information connected with it is sold, assigned, or transferred, in which case we would require the buyer, assignee, or transferee to treat your personal information in accordance with this Privacy Policy.

Also, your personal information may be disclosed to a third party if we are required to do so because of an applicable law, court order, or governmental regulation, or if such disclosure is otherwise necessary to support of any criminal or other legal investigation or proceeding here or abroad.

**Retention of personal information**

Your personal information will be stored for as long as the survey is available for completion, which is approximately six months from the start of the survey. After that time, your personal information will be deleted.

**Exercising of access rights and contact details**

You may request information about the personal information we collect and exercise your related access rights, including deletion of your personal information and withdrawal of your consent, by contacting [unsubscribe.bci@kpl-paris.com](mailto:unsubscribe.bci@kpl-paris.com)

Click here to acknowledge and access the questionnaire

**Supplementaty 3: Student Test results**

1. **Mexico – Adherence awareness & adherence support**

|  | **T0**  **n %** | | **T3**  **n %** | | | **T6**  **n %** | |
| --- | --- | --- | --- | --- | --- | --- | --- |
| **Do you think adherence is an important topic in your clinical practice?** | 15 | **6** | 14 | **6** | 15 | | **5,9** |
| **Do you think non-adherence can have an impact on patients' lives?** | 15 | **5,7** | 14 | **6** | 15 | | **5,8** |
| **Do you think adherence can fluctuate over time for each patient?** | 15 | **5,3** | 14 | **5,6** | 15 | | **5,7** |
| **Do you think healthcare professionals (HCPs) can influence patients' adherence?** | 15 | **5,6** | 14 | **5,9** | 15 | | **5,7** |
| **Do you have access to information/literature/training on adherence?** | 15 | **3,3** | 14 | **4,2** | 15 | | **4,5** |
| **Do you know the reasons why patients might not adhere to treatment?** | 15 | **3,6** | 14 | **4,4** | 15 | | **4,5** |
| **Do you address adherence during consultations with your patients?** | 15 | **4,2** | 14 | **4,9** | 15 | | **4,9** |

|  | **T0** | | **T3** | | **T6** | |
| --- | --- | --- | --- | --- | --- | --- |
| **Do you know how to assess levels of non-adherence in your patients and identify the underlying causes?** | 15 | **3,1** | 14 | **4** | 15 | **4,6** |
| **If yes, what percentage of your dyslipidemic patients are being assessed over a month?** | 15 | **41** | 14 | **58,2** | 15 | **64,7** |
| **total using Insight, over a period of a month, what percentage of screened patients would have been identified as** | | | | | | |
| Very high risk | 0 | 0 | 4 | **40** | 8 | **11,9** |
| High-risk | 0 | 0 | 4 | **23,8** | 8 | **26,3** |
| Medium risk | 0 | 0 | 4 | **12,5** | 8 | **13,1** |
| Low risk | 0 | 0 | 4 | **15** | 8 | **11,4** |
| Very low risk | 0 | 0 | 4 | **8,8** | 8 | **5,1** |
| **Does assessing the level of risk help you in your conversations with patients about treatment adherence ?** | 15 | **2,5** | 14 | **3,4** | 15 | **3,5** |
| **Are you following any instructions or practical tips to improve your style of communication?** | 15 | **2,6** | 14 | **4,6** | 15 | **4,7** |

1. **Thailand – adherence awareness & adherence support**

|  | **T0**  **n %** | | **T3**  **n %** | | | **T6**  **n %** | |
| --- | --- | --- | --- | --- | --- | --- | --- |
| **Do you think adherence is an important topic in your clinical practice?** | 27 | **5,74** | 26 | **5,42** | 25 | | **5,52** |
| **Do you think non-adherence can have an impact on patients' lives?** | 27 | **5,56** | 26 | **5,31** | 25 | | **5,2** |
| **Do you think adherence can fluctuate over time for each patient?** | 27 | **5,07** | 26 | **5** | 25 | | **4,92** |
| **Do you think healthcare professionals (HCPs) can influence patients' adherence?** | 27 | **5,33** | 26 | **5,19** | 25 | | **5,32** |
| **Do you have access to information/literature/training on adherence?** | 27 | **3,07** | 26 | **4,81** | 25 | | **5,32** |
| **Do you know the reasons why patients might not adhere to treatment?** | 27 | **3,59** | 26 | **4,42** | 25 | | **4,72** |
| **Do you address adherence during consultations with your patients?** | 27 | **4** | 26 | **4,77** | 25 | | **5** |

|  | **T0**  *n %* | | **T3**  *n %* | | **T6**  *n %* | |
| --- | --- | --- | --- | --- | --- | --- |
| **Do you know how to assess levels of non-adherence in your patients and identify the underlying causes?** | 27 | 2,44 | 26 | 4,27 | 25 | 4,52 |
| **If yes, what percentage of your dyslipidemic patients are being assessed over a month?** | 24 | 39,17 | 26 | 51,35 | 25 | 56,4 |
| **total using Insight, over a period of a month, what percentage of screened patients would have been identified as** | | | | | | |
| Very high risk | 2 | 0 | 23 | 9,91 | 24 | 8,13 |
| High-risk | 2 | 0 | 23 | 17,74 | 24 | 16,46 |
| Medium risk | 2 | 0 | 23 | 23,43 | 24 | 16,46 |
| Low risk | 2 | 0 | 23 | 20,61 | 24 | 11,67 |
| Very low risk | 2 | 0 | 23 | 14,04 | 24 | 15,21 |
| **Does assessing the level of risk help you in your conversations with patients about treatment adherence ?** | 14 | 2,64 | 18 | 3,44 | 7 | 3,71 |
| **Are you following any instructions or practical tips to improve your style of communication?** | 26 | 3,38 | 26 | 4,77 | 24 | 5,08 |
